# Optimization of the dosimetric leaf gap for use in planning VMAT treatments of spine SABR cases

**DOI:** 10.1002/acm2.12106

**Published:** 2017-06-02

**Authors:** Nigel D Middlebrook, Bess Sutherland, Tanya Kairn

**Affiliations:** ^1^ Genesis Cancer Care Queensland John Flynn Private Hospital Inland Drive Tugun Qld 4224 Australia; ^2^ Genesis Cancer Care Queensland Premion Place 39 White St Southport Qld 4215 Australia; ^3^ Genesis Cancer Care Queensland Wesley Medical Centre Suite 1 40 Chasely St Auchenflower Qld 4066 Australia; ^4^ School of Chemistry, Physics and Mechanical Engineering Queensland University of Technology 2 George St Brisbane Qld 4000 Australia; ^5^ Present address: Radiation Oncology Centres Gold Coast University Hospital Block C Lower Ground 1 Hospital Blvd Southport Qld 4215 Australia

**Keywords:** dosimetric leaf gap, multileaf collimators, RapidArc, spine SABR, treatment planning systems

## Abstract

The dosimetric leaf gap (DLG) is a beam configuration parameter used in the Varian Eclipse treatment planning system, to model the effects of rounded MLC leaf ends. Measuring the DLG using the conventional sliding‐slit technique has been shown to be produce questionable results for some volumetric modulated arc therapy (VMAT) treatments. This study therefore investigated the use of radiochromic film measurements to optimize the DLG specifically for the purpose of producing accurate VMAT plans using a flattening‐filter‐free (FFF) beam, for use in treating vertebral targets using a stereotactic (SABR, also known as SBRT) fractionation schedule. Four test treatments were planned using a VMAT technique, to deliver a prescription of 24 Gy in 3 fractions to four different spine SABR treatment sites. Measurements of the doses delivered by these treatments were acquired using an ionization chamber and radiographic film. These measurements were compared with the doses calculated by the treatment planning system using a range of DLG values, including a DLG identified using the conventional sliding‐slit method (1.1 mm). An optimal DLG value was identified, as the value that produced the closest agreement between the planned and measured doses (1.9 mm). The accuracy of the dose calculations produced using the optimized DLG value was verified using additional radiochromic film measurements in a heterogeneous phantom. This study provided a specific initial DLG (1.9 mm) as well as a film‐based optimization method, which may be used by radiotherapy centers when attempting to commission or improve an FFF VMAT‐based SABR treatment programme.

## 1 INTRODUCTION

Stereotactic ablative radiosurgery (SABR, also known as stereotactic body radiotherapy or SBRT) has been shown to be effective for treating tumors in and around the vertebra.^1^, ^2^ These “spine SABR” treatments require the use of a small number of treatment fractions (typically 1–4) to deliver a relatively high dose of radiation (typically 12–27 Gy).^3^ In order to minimize the time taken to deliver these high‐dose fractions, especially for patients who may be suffering pain and discomfort due to vertebral metastases, treatments can be delivered using high dose rate modes. Dose rates of up to 2200 MU/min can be achieved by using flattening filter free (FFF) modes on contemporary linacs. Compared to the maximum dose rates of 600 MU/min for flattened 6 MV and 10 MV beams, the FFF beams greatly reduce the beam‐on time.

The Spine SABR target volumes are generally irregular in shape due to the location and geometry of the targeted vertebra as well as the importance of sparing the spinal cord, which abuts or penetrates the target volume. Treatment planning using inversely optimized volumetric modulated arc therapy (VMAT) techniques, can result in very steep dose gradients (greater than 12%/mm^4^) between the targeted vertebra and the spinal cord. To produce the complex dose distributions required to achieve that spinal cord sparing while adequately treating the targeted vertebra, VMAT uses moving multileaf collimators (MLCs), with simultaneously varying dose rates and gantry speeds.^5^ These complex, dynamic systems present numerous opportunities for dose uncertainties.

The AAPM Task Group‐101 report highlighted the accuracy required in treatment planning for SABR treatments,^6^ and recommended rigorous testing of the TPS dose calculation accuracy including end‐to‐end tests. Accurate calculations of dose and dose gradients are especially important for treatments where ablative doses of radiation are delivered to targets in close proximity to critical structures, such as spine SABR treatments. Dose calculation accuracy is known to be detrimentally affected by the use of suboptimal beam configuration data in the radiotherapy treatment planning system (TPS)^7^, ^8^ and by the inappropriate handling of simplifications in the TPS model.^9^, ^10^, ^11^

For example, Varian Eclipse TPS (Varian Medical Systems, Palo Alto, USA) simplifies the modelling of the physical geometry of the MLC leaves, omitting physical characteristics such as the rounded leaf‐ends. To overcome this, Eclipse allows the user to define a specific parameter, the dosimetric leaf gap (DLG), which defines the difference between the physical round leaf end and the straight edge model of the TPS.^9^ The value of the DLG is applied when calculating dose for modulated radiotherapy (including VMAT) treatment plans, as a retraction between the planned and calculated MLC positions. The DLG parameter is one of a few values that needs to be modified by the user when configuring the Varian Eclipse anisotropic analytical algorithm (AAA).^10^

Measurement of the DLG is performed by the sliding‐slit test (as described in Varian Medical Systems’ documentation^10^). This method produces a single DLG value per energy, which is applied in the Eclipse TPS to all leaf pairs irrespective of MLC leaf width.^9^ While some studies have identified good agreement between planned and measured doses when using the DLG value measured using the standard sliding‐slit test,^12^, ^13^ other authors have identified substantial discrepancies.^11^, ^14^ For example, Szpala et al.^11^ and Kielar et al.^14^ elected to optimize the DLG value using clinical VMAT plans after they observed that the DLG value measured using the sliding slit test produced unreliable results when used to calculate clinical VMAT plans. These authors recommended careful testing for dosimetric accuracy for irradiating small targets, especially those used for SABR.

Similarly, both Szpala et al.^11^ and Kumaraswamy et al.^9^ found that the single DLG value used in Eclipse should be considered an estimate only; the optimal DLG for each MLC leaf varies with the distance from the central‐axis and with the position of the opposite leaf. Due to the differences between the field sizes and complexity of MLC motion required when treating different anatomical sites,^15^ the DLG can be expected to vary with anatomical treatment site and treatment modality.

Previous examinations of the Varian DLG have focused on treatments with standard (nonstereotactic) fractionation, planned for the brain,^9^, ^11^, ^14^ prostate,^9^, ^12^ head and neck,^9^, ^12^ and AAPM Task Group 119 standard volumes (average prostate and simplified spine).^13^, ^14^ Some of these studies have suggested that the DLG values that are required to accurately calculate dose for FFF modalities are especially different from the DLG values that are obtained using the sliding slit method.^14^, ^16^ It is therefore important to specifically evaluate and optimize the DLG that is used when calculating dose for hypo‐fractionated SABR treatments that use FFF VMAT beams.

This study therefore demonstrates the use of radiochromic film measurements to investigate the optimal DLG for use when treating spine SABR cases using a VMAT technique, with an FFF beam, in order to provide a specific initial DLG as well as a film‐based optimization method, which may be used by radiotherapy centers when attempting to commission or improve an FFF VMAT‐based SABR treatment programme.

## 2 MATERIALS AND METHODS

### 2.A Test treatment plans

The prescription used for the clinical test spine SABR treatment plans was 24 Gy, to be delivered in 3 fractions of 8 Gy. This prescription was selected with reference to literature^16^ including the Canadian^17^ and ASTRO guidelines.^18^ All spinal target volumes were contoured according to the International Spine Radiosurgery Consortium Consensus Guidelines for Target Volume Definition in Spinal Stereotactic Radiosurgery 2011^1^ and ROTG 0631.^2^ The beam arrangement for all plans used two counter‐rotating 360° arcs delivered on a Varian TrueBeam linac with a Millennium MLC, operating in 6 MV FFF photon mode. The maximum achievable dose rate for this beam was 1400 MU/min. The characteristics of these treatment plans are summarized in Table 1.

**Table 1:** Overview of the properties of the four VMAT spine SABR test treatment plans used in this study

Property	Case 1		Case 2		Case 3		Case 4	
Arc number	1	2	1	2	1	2	1	2
MU	1777	2911	2633	1682	2571	1551	2158	2107
X field size (mm)	90	56	49	38	67	42	67	64
Y field size (mm)	52	91	40	49	45	66	45	64
PTV‐cord separation (mm)	1.6		1.6		1.3		1.3	

### 2.B DLG optimization: Homogeneous phantom

An initial DLG value was measured using Varian supplied DICOM files for the sliding slit method. This test involves measuring the ionization at central axis by varying slit sizes (2, 4, 6, 10, 14, 16, 20 mm) as they move across a field with constant speed. A linear fit to determine the intercept provides a result for the DLG. Control points are set every 10 mm.

Based on the results of the standard sliding‐slit measurement described above, the value of 1.1 mm for DLG was initially used for dose calculation of spine SABR plans. Dose was calculated using Eclipse AAA dose calculation algorithm version 11.0.31. Verification plans were created for a Blue Solid Water (Standard Imaging, Wisconsin, USA) phantom with a dose grid size of 2.5 mm. The Varian IGRT couch was included in the dose calculation. The phantom was set up on the treatment couch for three separate measurements per plan: point dose measurements using a CC13 ionization chamber (IBA Dosimetry, Bartlett, USA), and two‐dimensional dose planes through the isocenter for both the transverse and coronal planes, using Gafchromic EBT3 film (International Speciality Products, Wayne, USA). The chamber dose values calculated by Eclipse were determined from a point dose measurement at the effective point of measurement for the CC13. Although the chamber was positioned within a high‐dose plateau region of the PTV, dose gradients of up to 5%/mm existed in this region.

The transverse plane was chosen for the film measurements as best represents the clinical aim of dose sparing of the spinal cord. Dose agreement was evaluated separately for each arc using the coronal planes and evaluated for each entire treatment plan using the transverse planes.

Film calibration irradiations were also performed with the 6FFF beam at full dose rate of 1400 MU/min. Film preparation, calibration and analysis was performed as per the method outlined by Kairn et al.^19^ Regions of interest used for the calibration films were approximately 5 mm by 5 mm. All films were scanned before and after irradiation using the Epson v800 (transmission mode), 72 DPI, 48 bit color. The red channel only was used for analysis. SNC Patient V6.1 (Sun Nuclear Corporation, Melbourne) was used to perform film vs planned dose comparisons using gamma analysis^20^ (absolute dose criteria 3%/1.5 mm) and quantitatively examining the agreement between the dose profiles.

The DLG value was then iteratively altered, and each Spine SABR verification plan was calculated and compared to the measured dose distribution. This optimization process continued until the optimal DLG value was identified as the value that resulted in the best overall agreement between calculated and measured dose, for the four test plans.

### 2.C DLG verification: Heterogeneous phantom

The suitability of the optimized DLG value was evaluated in an inhomogeneous phantom, the IMRT Thorax phantom (CIRS Inc, Norfolk, USA), using a fine (1 mm) dose calculation grid resolution. Only two DLG values were used when calculating the Spine SABR plans on the IMRT Thorax phantom – the initial 1.1 mm and the optimal 1.9 mm value. As these measurements in the transverse plane were used to evaluate the sparing of the spinal cord region as well as the accurate treatment of the planned high‐dose (vertebral) region, both arcs from each treatment were delivered to each piece of film. This represents a single fraction treatment dose.

## 3 RESULTS

### 3.A DLG optimization: Homogeneous phantom

**Figure 1:**
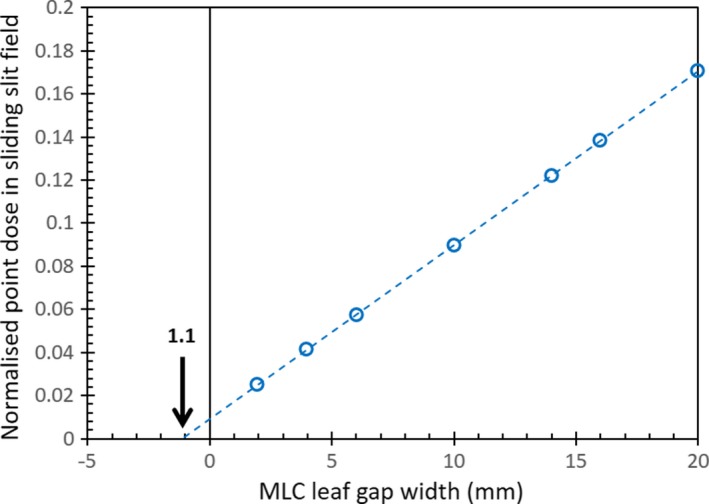
DLG measurement using sliding‐slit method. A DLG of 1.1 mm was obtained by extrapolating the results obtained at different sliding slit widths, to identify the theoretical slit width that would produce a measurement of zero dose.

The DLG value for the 6 MV FFF beam with Millenium‐120 MLC was found to be 1.1 mm using the sliding slit method, as shown in Fig. 1.

**Figure 2:**
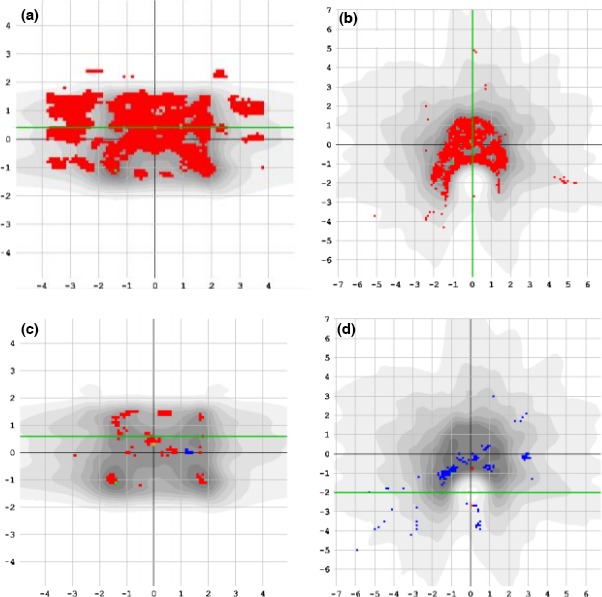
Typical results of comparing dose measured with film against dose calculated by the treatment planning system using the sliding‐slit‐based DLG (1.1 mm), for (a) case 4, arc 2, in the coronal plane and (b) case 3, both arcs, in the transverse plane. Results using optimization‐based DLG (1.9 mm), for (c) case 4, arc 2, in the coronal plane and (d) case 3, both arcs, in the transverse plane. Red pixels indicate that the measured dose exceeded the planned dose sufficiently for the points to fail a gamma evaluation at 3%, 1.5 mm.

Using the DLG value identified using the sliding‐slit method (1.1 mm), initial results of the Spine SABR test plans show large differences between measured and planned dose distributions (see Figs. 2(a) and 2(b)).

**Figure 3:**
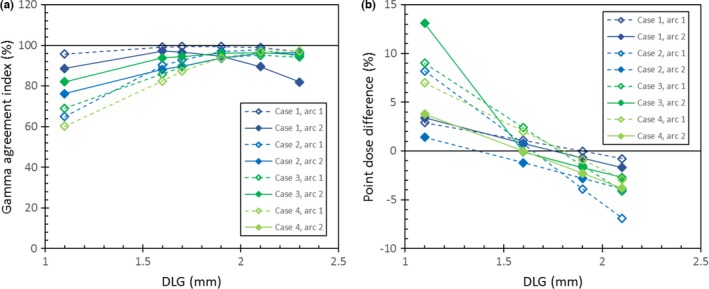
Results of comparing dose measured with (a) film and (b) ionization chamber against dose calculated by the treatment planning system for various DLG values. Close agreement between the measured and planned doses is indicated by (a) gamma agreement indices close to 100%, for the film measurements, and (b) dose differences close to 0%, for the ionization chamber measurements. Point dose difference is defined as (measured – TPS calculated)/measured.

Figures 3(a) and 3(b) summarize the gamma agreement rates for the range of DLG values trialled for each of the Spine SABR test plans. From these results, the optimal DLG from the film measurements for the FFF Spine SABR is in the range 1.9–2.1 mm. The chamber measurements are shown in Fig. 3(b) – the optimal DLG is in the range 1.6–1.9 mm.

A value of 1.9 mm was therefore selected as the optimal DLG for use when planning FFF VMAT spine SABR treatments. Figures 2(c) and 2(d) shows the same fields as Figs. 2(a) and 2(b), re‐calculated using the optimal DLG value of 1.9 mm.

### 3.B DLG verification: Heterogeneous phantom

**Figure 4:**
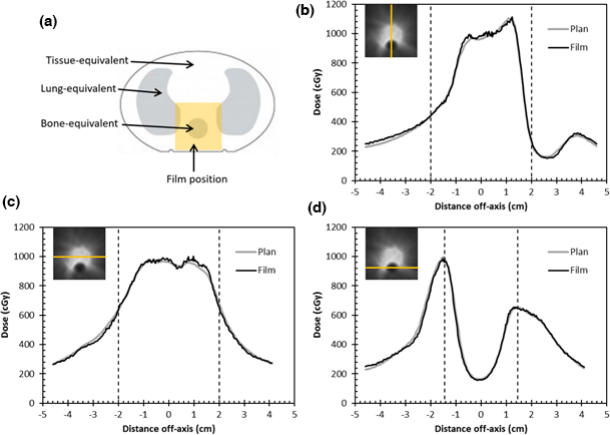
Typical results of evaluating dose profiles through a transverse plane in the heterogeneous (thorax) phantom (case 2, both arcs): (a) film position (yellow square) in heterogeneous phantom; (b) anterior‐posterior profiles through PTV and spinal cord; (c) lateral profiles through PTV; (d) lateral profiles through spinal cord. Dose profiles were obtained from film measurement (thinner, black lines) and from dose calculated by the treatment planning system using the optimization‐based DLG (1.9 mm) on a 1 mm dose calculation grid. Insets show profile locations and vertical dotted lines indicate the locations of bone‐tissue interfaces.

Table 2 summarizes the gamma agreement results for each of the four test cases delivered to the thorax phantom. Figure 4 shows profiles comparing dose plane from treatment plan and film measurement in CIRS thorax phantom.

**Table 2:** Gamma agreement indices (percentage of points passing a gamma evaluation using 3%, 1.5 mm criteria) resulting from comparing the dose measured using film in a transverse plane through the heterogeneous (thorax) phantom against the dose calculated in the same plane using the treatment planning system with the sliding‐slit‐based DLG (1.1 mm) and the optimization‐based DLG (1.9 mm)

DLG (mm)	Case 1	Case 2	Case 3	Case 4
1.1	64.6	70.1	76.7	61.3
1.9	95.6	99.2	96.4	98.5

## 4 DISCUSSION

The DLG value identified in this study using the sliding‐slit method for the 6 MV FFF beam (1.1 mm) lies between the published values reported by Glide‐Hurst et al. (1.16 mm^13^) and Chang et al. (0.71 mm^12^). However, the results of this study are also in agreement with the observations of Kielar et al.;^14^ the conventional sliding‐slit method does not produce clinical treatment plans that show good agreement between planned and measured doses for VMAT treatments delivered using a FFF beam. Data shown in Table 2 confirm that use of the DLG obtained through an optimization process, using VMAT spine SABR test treatment plans (1.9 mm), results in treatment plans that are substantially more dosimetrically accurate than use of the DLG obtained from the conventional sliding‐slit measurement (1.1 mm).

The Eclipse AAA beam model used in this study was commissioned using data for field sizes ranging from 3 × 3 cm^2^ to 40 × 40 cm^2^.^10^ Although data for smaller field sizes is usually measured during linac commissioning, it is not required for commissioning of the beam model within Eclipse.^12^

The DLG is used in the Varian Eclipse treatment planning system as an approximation factor to reduce the dosimetric calculation uncertainty arising from the use of a simple MLC model with straight leaf ends. Conventionally, the DLG is measured using vendor‐supplied DICOM plans that produce a sliding‐slit with 13 control points, where the MLC leaves move at the same speed, in one direction, with a constant dose rate.^10^ This broadly approximates an IMRT delivery, where the MLC leaves move in the same direction, from one side of the field to the other, albeit at different speeds.

By contrast, VMAT treatment deliveries are much more complex. Each VMAT arc typically uses 178 controls points, with MLC leaves undergoing frequent changes in direction. Adjacent MLC leaves may move in opposite directions to each other and at different speeds. The dose rate is also modulated and defined for each control point. A single point measurement using the sliding slit method does not replicate the complex MLC movements such as those in a VMAT treatment for a spine SABR case.

It is therefore unsurprising that determination of the appropriate DLG value for clinical use in planning VMAT treatments should require the use of more complex plans than the sliding‐slit, evaluated using more sophisticated measurements than a point dose. Optimization of the DLG for VMAT treatments should involve the use of treatment plans that are representative of intended clinical use of the beam model, with measurements completed using accurate, high‐resolution two‐dimensional dosimeters.^9^, ^11^, ^13^, ^14^

In this study, radiochromic film was shown to produce results that were sufficiently sensitive to DLG variation for use in DLG optimization, although verification using a second dosimeter (such as an ionization chamber) may be advisable (see Fig. 3). The radiochromic film used in this study also provided accurate, high‐resolution measurements that allowed the suitability of the optimized DLG value to be verified, when dose was calculated at a high resolution and the test treatments were delivered to a heterogeneous phantom (see Fig. 4). Estimated measurement uncertainties affecting the use of radiochromic film for radiotherapy dosimetry range from 0.55%^21^ to 4%.^22^ It is therefore important to independently evaluate uncertainties when commissioning any radiochromic film dosimetry system that is used to optimize beam configuration values, such as the DLG.

Figure 2 show example results where film was used to evaluate the accuracy of the planned dose calculation in the coronal and transverse planes. Film measurements are frequently undertaken using the coronal plane,^11^, ^13^, ^14^ where accurate and reproducible measurement set‐ups are easy to achieve by sandwiching films flat on the linac couch between phantom slabs. However, when examining or verifying the dose distribution for Spine SABR plans, it is important to evaluate dose in the transverse plane (as shown in Figs. 2(b) and 2(d))) because the accuracy of the high dose gradient between the PTV and spinal cord is a critical treatment parameter, affecting the safety and clinical acceptability of the treatment plan.^4^ Additional care must be taken, when making film measurements in the transverse plane, as small air gaps between the film and the phantom, or small rotations or offsets in the phantom setup could cause large differences in measured dose distributions.

The results shown in Fig. 3 confirm the importance of optimizing the DLG using a range of clinically likely test treatments. For this study, the test treatment volumes were thoracic and lumbar vertebral bodies, with and without left and right pedicles, and the corresponding treatments were designed with a range of different field sizes and collimator angles. Figure 3 shows that the particular values of the DLG that gave the closest agreement between the planned and measured doses differed between plans and between measurement techniques. We have adopted the optimal DLG of 1.9 mm for the 6FFF beam model for use in our clinic, for treatment of spine SABR cases. We have not yet investigated the application of this optimal DLG to SABR planning for other anatomical sites. The identification of a DLG value that is optimal for an entire class of plans (for a specific treatment modality, used to treat a specific anatomical site) evidently requires the use of different examples of the specific anatomical site to be treated.

## 5 CONCLUSIONS

This study used an evaluation of DLG suitability for four spine SABR test treatment plans to confirm that the DLG identified using the conventional sliding‐slit method does not produce clinical treatment plans that show good agreement between planned and measured doses for VMAT treatments delivered using a FFF beam.

Based on the results of this study, the following general recommendations can be made, for optimizing the DLG for use in planning spine SABR (or any other) VMAT treatments:

1. A range of clinically likely test treatments should be used when optimizing the DLG;
2. The suitability of each tested DLG value should be evaluated against measurements made using an accurate high‐resolution dosimeter, such as radiochromic film;
3. The film measurement plane and position should be selected in order to provide clinically relevant results (e.g., a coronal plane through each PTV as well as a transverse plane through each PTV and spinal cord, for spinal SABR);
4. For treatments planned for delivery to heterogeneous anatomy (e.g., spine, lung, head and neck) and for treatments where especially steep dose gradients are required (e.g., spine SABR, cranial stereotactic radiosurgery), the accuracy of the calculated dose should be verified using measurements in heterogeneous phantoms and calculations at fine dose grid resolutions;
5. Care should be taken at all stages of the measurement and analysis (including copying the treatment plan to the measurement phantom, setting up the film within the phantom, setting up the phantom for measurement, and scanning and analysing the films), because this measurement is used to help determine a parameter in the treatment planning system beam configuration data (the DLG), and consequently errors introduced at this stage have the potential to affect a large number of patients.

## CONFLICT OF INTEREST

The authors have no conflicts of interest to disclose.
